# To the Editors of the Medical and Physical Journal

**Published:** 1799-05

**Authors:** Thomas Beddoes


### *To the Editors of the Medical and Physical Journal.*

GENTLEMEN,

AN important error has been difcovered in part of the MS. of the "*Weft Country Contributions*;" and I fhould be much obliged to you to inform the poffeffors of that work, that at p. 381. l. 9, they fhould read "*three grains*," inftead of "*nine grains*."

I am, Gentlemen, your's, &c.

THOMAS BEDDOES.

